# Whole-Body Cryotherapy Reduces Systemic Inflammation in Healthy Adults: Pilot Cohort Study

**DOI:** 10.2196/60942

**Published:** 2024-11-22

**Authors:** Elizabeth Chun, Richard Joseph, Rachele Pojednic

**Affiliations:** 1 Restore Hyper Wellness Austin, TX United States; 2 Department of Statistics Texas A&M University College Station, TX United States; 3 Department of Health and Human Performance Norwich University Northfield, VT United States; 4 Stanford Lifestyle Medicine Stanford Prevention Research Center Stanford University School of Medicine Palo Alto, CA United States

**Keywords:** cold therapy, C-reactive protein, fasting glucose, HbA1c, inflammation, lipid metabolism, whole-body cryotherapy, cryotherapy, retrospective, reactive protein, biomarker, adult, systemic inflammation

## Abstract

**Background:**

Chronically elevated inflammation is implicated in many conditions, including obesity, metabolic syndrome, and cardiovascular disease, and has been associated with increased mortality risk. Whole-body cryotherapy (W-BC) is a promising modality to treat inflammation with demonstrated benefits for clinical subpopulations including those with arthritis, obesity, and type 2 diabetes. However, it is unclear whether the benefit from W-BC extends to healthy individuals prior to chronic disease–related inflammation. In addition, the long-term durability of W-BC effect is unknown.

**Objective:**

This study investigates the inflammatory response to W-BC in healthy adults with a biomarker of inflammation, high-sensitivity C-reactive protein (hsCRP), and clinical biomarkers of metabolism including fasting glucose, hemoglobin A_1c_ (HbA_1c_), low-density lipoprotein (LDL) and high-density lipoprotein (HDL), and triglycerides.

**Methods:**

Fifteen individuals (n=9 female) participated in frequent recreational W-BC (3 minutes of cold exposure at –110 ℃) over approximately 9 months and had blood draws at baseline plus follow-up visits. Biomarkers were modeled as linear functions of W-BC sessions received in the month prior to blood draw.

**Results:**

The mean amount of W-BC received was 6.78 (SD 4.26) times per month with the cumulative total ranging from 13 to 157 W-BC sessions over the course of the study. On average, participants completed 1-2 sessions per week throughout the intervention. The number of W-BC sessions were associated with decreased hsCRP (–0.14 mg/L in hsCRP per W-BC session; *P*<.01) and with durability of up to 9 months. Increased W-BC was also associated with a downward trend in fasting glucose. This trend failed to reach significance at 1 month (–0.73 mg/dL in fasting glucose per W-BC session; *P*<.10) but was significant for 2- and 3-month windows (*P*<.05). HbA_1c_ was increased significantly after 9 months (*P*<.01); however, the change occurred within normal ranges (difference=0.13% and <5.7%) and was not clinically significant. There was no association between W-BC and LDL cholesterol, HDL cholesterol, or triglycerides (*P*>.10), although LDL trended lower over the time period examined (*P*=.07).

**Conclusions:**

These results suggest that W-BC beneficially impacts systemic inflammation by lowering hsCRP levels in healthy individuals and may also have some modulating effect on fasting glucose.

## Introduction

Chronic systemic inflammation is associated with aging [1] and aging-related diseases [2] including increased cardiovascular disease, cancer, and overall mortality [3,4]. Systemic inflammation is also implicated in metabolic conditions such as diabetes, overweight, and the cluster of conditions collectively known as metabolic syndromes [5]. As populations age and chronic conditions increase worldwide [2], identifying nonpharmacologic therapies to proactively treat inflammation in healthy individuals has become paramount.

One potential approach is cold therapy, which has been examined for acute and disease-specific inflammation [6]. While cold therapy has historically involved either localized treatment or cold water immersion, in recent years cryotherapy machines use air chambers that reach below –100 ℃ [7]. Multiple forms of cryotherapy exist, one of which is whole-body cryotherapy (W-BC) in which a person is completely enclosed in a cryotherapy chamber. In sports medicine, W-BC has been shown to reduce inflammation markers including interleukin-6 (IL-6) and C-reactive protein (CRP) [8]. Separately, in inflammatory diseases such as rheumatoid arthritis, W-BC has been used to reduce pain and disease activity [9]. W-BC has been shown to decrease CRP [10] and proinflammatory cytokines IL-6 and resistin, while increasing the anti-inflammatory cytokine IL-10 in men with obesity [11]. W-BC has also demonstrated reductions in CRP in postmenopausal women with type 2 diabetes [12]. Although these results are promising, there remains a crucial gap in long-term study of inflammation specifically in healthy individuals as further value could be extracted if benefits extend prior to disease onset. Moreover, the chronic, rather than acute, impact of W-BC on inflammation is insufficiently studied as most literature focuses on the time frame immediately following treatment [7]. Thus, the durability of such an effect is not well established. This study aimed to analyze the impact of W-BC on systemic inflammation and related markers of metabolism in healthy adults within a free-living environment. To understand the longer-term effect, durability analysis was also performed. It was hypothesized that W-BC would have a beneficial impact on inflammation in healthy adults, possibly mediated via an anti-inflammatory effect of lowering blood glucose.

## Methods

### Participants

The study cohort was composed of healthy adults older than 18 years whose deidentified data were abstracted from client records for research analysis. Data were collected from individuals who had been screened for contraindications and exclusion criteria as previously reported [13]. Briefly, participants were excluded if they reported a diagnosed chronic disease, had untreated high or low blood pressure, or were pregnant or breastfeeding. All participants completed a consent that allowed data to be used for research purposes.

### Ethical Considerations

Data were examined retrospectively and determined to be exempt by the Norwich University institutional review board and research ethics committee and informed consent was waived (HHS IORG #0004914; institutional review board no. 00005859; date of registration: April 30, 2023).

### Intervention Protocol

Participants were followed for an average of 8.55 months (SD 3.13; median 6.77, IQR 6.77-9.73), with total follow-up duration ranging from 5 to 13 months for those who had at least 1 follow-up visit. During this time, they frequently participated in recreational W-BC at a commercially operated studio (Restore Hyper Wellness). The mean amount of W-BC received was 6.78 (SD 4.26) times per month with the cumulative total ranging from 13 to 157 W-BC sessions over the course of the study. On average, participants completed 1-2 sessions per week throughout the intervention. Participants were encouraged to engage in regular physical activity and received guidance on healthy dietary patterns, although these behaviors were not monitored.

### Whole-Body Cryotherapy Protocol

Participants were escorted to a private changing room where they removed personal clothing and put on a provided hat, socks, slippers, mask, and gloves, as well as a towel or robe. Once changed, they were escorted to the cryotherapy chamber for 3 minutes of cold exposure at –110 ℃. Chambers were electrically cooled using a main cooling unit in a full body chamber (Zimno Tech). Participants were observed by an employee trained in administering the protocol and could exit the chamber at any time. At the completion of 3 minutes, the chamber door was opened and the participants exited, returned to the private changing room, and redressed in their own clothing.

### Blood Biomarkers

Blood was drawn at baseline and then incrementally at follow-up visits. Participants were asked to fast for 8-12 hours prior to blood draw. On average, there were 2.13 (SD 2.59) follow-up visits with a mean time of 170 (SD 84) days between blood draws. The minimum time between blood draws was 17 days, and the maximum was 292 days. Blood biomarkers measured include serum high-sensitivity C-reactive protein (hsCRP), fasting glucose, hemoglobin A_1c_ (HbA_1c_), low-density lipoprotein (LDL) and high-density lipoprotein (HDL), and triglycerides. All blood was drawn from the median cubital vein with a 20-gauge intravenous catheter needle. Drawn samples were sent to Quest Diagnostics for analysis. hsCRP was analyzed with an immunoturbidimetric assay (test code 10124); fasting glucose, serum lipids, and triglycerides were analyzed with spectrophotometry (test codes 6881 and 7600, respectively); and HbA_1c_ was analyzed with an enzymatic assay (test code 496). For hsCRP, the reporting range by Quest Diagnostics was 0.3-10 mg/L, with values outside the range being listed as <0.3 or >10 mg/L. There were no identified thresholds for fasting glucose, HbA_1c_, or lipids.

### Statistical Analysis

All data analysis and statistical modeling were carried out in R (version 4.3.1) [14]. Data processing was performed using tidyverse packages including readr and readxl for file opening; dplyr, hms, lubridate, tibble, and stringr for wrangling; and ggplot2 for visualizations [15]. Two data points were missing a parsable date and those records were removed. Out of range hsCRP measurements were included as their threshold value, that is, <0.3 was included as 0.3.

Statistical significance was defined as a *P*<.05 threshold while anything failing to meet that threshold but with *P*<.10 was noted as being mild association and of possible interest. Paired *t* tests on all complete pairs for a given analyte were used to compare start and end measurements for all biomarkers in Table 1.

**Table 1:** Summary of study cohort—demographics and blood biomarkers.

Variable	Start mean (SD)	End mean (SD)	*P* value^a^
N	15	13	N/A^b^
Sex (female/male)	9 females/6 males	8 females/5 males	N/A
Age (years)	48.60 (8.50)	48.50 (8.31)	N/A
HbA_1c_ (%)	5.12 (0.27)	5.25 (0.29)	.008^c^
Fasting glucose (mg/dL)	96.00 (15.96)	98.31 (12.87)	.93
hsCRP^d^ (mg/L)	3.39 (3.31)	1.88 (1.88)	.03^c^
LDL^e^ (mg/dL)	121.07 (30.22)	112.00 (37.05)	.07
HDL^f^ (mg/dL)	57.87 (12.35)	58.92 (13.47)	.76
Triglycerides (mg/dL)	113.47 (54.58)	119.92 (63.46)	.81

^a^*P* values calculated using paired 2-tailed *t* tests on all complete pairs for a given analyte.

^b^N/A: not applicable.

^c^*P*<.05.

^d^hsCRP: high-sensitivity C-reactive protein.

^e^LDL: low-density lipoprotein.

^f^HDL: high-density lipoprotein.

**Figure 1:**
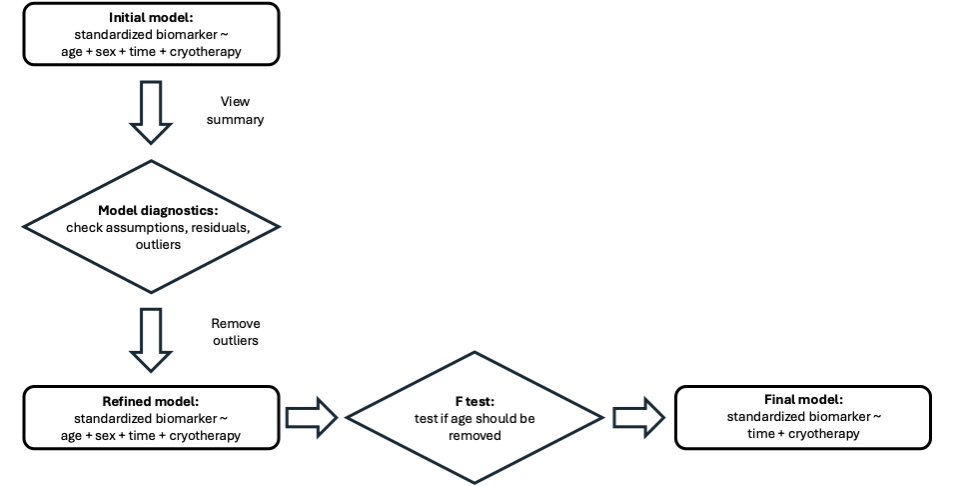
Statistical analysis flowchart. Diagram of study statistical methods showing each modeling step. For durability testing, the final model structure, namely, analyte regressed on days from study start and number of W-BC sessions, was used to fit models on different time windows. In particular, 1- to 3-week, 1- to 3-month, 6-month, and 9-month windows were tested. The false discovery rate method was used to correct for multiple testing.

For both the primary analysis (W-BC impact on hsCRP) and the secondary analysis (W-BC impact on biomarkers of metabolism) multiple linear regression models were constructed. An initial model was fit with the analyte of interest standardized by the participant-specific mean as the response, and the predictors were age, sex, days from study start, and number of W-BC sessions in the past 30 days (1 month). After inspection of model diagnostics for violations of the linear modeling assumptions, outliers were removed based on a Cook’s distance threshold of 4/n where n is the sample size. A refined model was then fit without outliers and inspected again for model diagnostics. Finally, the reduction method F test was used to determine whether a reduced model that removed age and sex was preferred. This was done because age and sex were not found to be significant in any of the initial models. Based on the F test results, the final model was chosen (Figure 1).

## Results

### Participants

Fifteen participants (female: n=9) were included in the analysis (Table 1). Ages ranged from 32 to 59 years. Average weight at baseline was 84.97 (SD 14.61) kg. Ethnicity or race and height were not recorded. Two were lost to follow-up and an intention-to-treat statistical model was used.

### Inflammation and hsCRP

**Figure 2:**
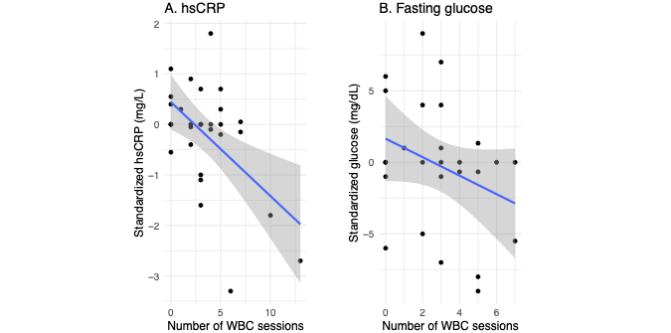
Model results. (A) Impact of W-BC on hsCRP counting W-BC sessions in the month prior to serum measurement. The y-axis shows hsCRP values standardized by subtracting subject specific means, while the x-axis is the number of W-BC sessions. The final linear regression was fit on 29 observations from 15 subjects (9 females/6 males); *P*<.01. (B) Impact of W-BC on fasting glucose modeled in the same fashion as hsCRP in (A). The final linear regression was fit on 26 observations from 14 subjects (9 females/5 males); *P*=.07. hsCRP: high-sensitivity C-reactive protein; W-BC: whole-body cryotherapy.

The number of W-BC sessions in the month prior to blood measurement was associated with a statistically significant decrease in hsCRP (*P*<.01). The final linear model for hsCRP was fit on 29 observations from 15 participants. Neither age nor sex was associated with changes in hsCRP and was not included in the final model (Figure 2A). The effect size was –0.1413 mg/L in hsCRP per W-BC session (95% CI –0.2289 to –0.0537).

To account for possible time-related confounding, time was adjusted for by including days since baseline launch day. Time was found to be a significant predictor of hsCRP although with a very small effect size (*P*<.01; effect size=–0.0047 mg/L, 95% CI –0.0066 to –0.0028). Importantly, even with time in the model, the number of W-BC sessions was still strongly significant. In addition, W-BC sessions had a larger effect size being –0.1413 mg/L as compared with –0.0047 mg/L for time. If scaled to 1-month windows, the effect of W-BC was still larger than time. Namely, multiplying days by 30 and W-BC sessions by the monthly average of 6.78 gives monthly effects sizes of –0.9579 mg/L and –0.1409 mg/L for W-BC and time, respectively. Thus W-BC sessions have a statistically significant impact on reducing hsCRP while also having a larger effect as compared with time (Table 2).

**Table 2:** Results for primary (hsCRP) and secondary (fasting glucose) analyses.

Response metric and predictor		Effect size^a^	95% CI	Scaled effect size^b^	*P* value
**hsCRP** ^c^ **(mg/L)**					
	W-BC^d^	–0.1413	–0.2289 to –0.0537	–0.9579	.0027^e^
	Time	–0.0047	–0.0066 to –0.0028	–0.1409	.00004^e^
**Fasting glucose (mg/dL)**					
	W-BC	–0.7337	–1.5236 to 0.0562	–4.9743	.0672
	Time	0.0114	–0.0008 to 0.0237	0.3423	.0663

^a^Effect size given as units of analyte per W-BC session (W-BC) or day (time), for example, for hsCRP, the effect size is –0.1413 mg/L in hsCRP per W-BC session.

^b^Scaled to 1 month by multiplying coefficients by 30 days for time and 6.78 for W-BC (mean amount of W-BC received per month).

^c^hsCRP: high-sensitivity C-reactive protein.

^d^W-BC: whole-body cryotherapy.

^e^*P*<.05.

**Figure 3:**
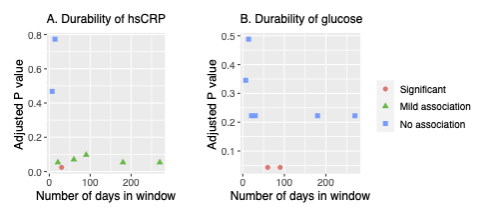
Durability testing. (A) Durability of whole-body cryotherapy (W-BC) effect on hsCRP measured by fitting linear models on a range of window sizes: 1-3 weeks, 1-3 months, 6 months, and 9 months. Models first reached mild association (*P*<.1) at 3 weeks which persisted up to the 9-month window. (B) Durability of W-BC effect on fasting glucose modeled in the same fashion as hsCRP in (A). Only 2- and 3-month models were significant (*P*<.05). Overall trend suggests an association that takes time to appear and tails off for very long windows. hsCRP: high-sensitivity C-reactive protein.

To elucidate the durability of the observed effect, sequential time windows were tested (Figure 3A). Windows less than 3 weeks were not associated (*P*>.10) with hsCRP. At 3 weeks, a mild association appeared (*P*>.10), and at 1 month the association was significant even after adjusting for the multiple testing windows (*P*<.05). For windows of 2 months or longer, the association remained, though only mildly (*P*>.10). Because participants were tracked only for around 9 months on average, longer windows were not constructed.

### Biomarkers of Metabolism

The number of W-BC sessions in the prior 1 month failed to reach the threshold for statistical significance (*P*=.07). The final linear model was fit on 26 observations of fasting glucose from 14 participants. Neither age nor sex was associated with fasting glucose and was not included. The estimated effect size was –0.7337 mg/dL in fasting glucose per W-BC session (95% CI –1.5236 to 0.0562) meaning more W-BC was mildly associated with a small decrease in fasting glucose (Figure 2B). Time indicated a possible small increase in fasting glucose over time although failed to reach significance (*P*=.06; effect size=0.0114, 95% CI –0.0008 to 0.0237). HbA_1c_ did increase slightly after 9 months (*P*<.01); however, the change was quite minimal and within normal ranges (difference=0.13% and <5.7% at both time points). Only hsCRP was reduced significantly (*P*=.03) in before versus after measurements.

Durability results revealed that slightly longer time windows may be more appropriate for studying fasting glucose. After correction for multiple testing using the false discovery rate method, the shorter 1-month window was not statistically significant (adjusted *P*>.05). However, windows of 2 and 3 months (60 and 90 days) showed a significant association between W-BC and decreased fasting glucose (adjusted *P*<.05; Figure 3B).

For the lipid biomarkers, namely, LDL cholesterol, HDL cholesterol, and triglycerides, no association with W-BC was found (*P*>.10), although LDL trended lower over the time period examined (*P*=.07).

## Discussion

### Principal Findings

This study investigated long-term physiologic effects associated with inflammation and aging in healthy adults by examining hsCRP and biomarkers of metabolism as a function of W-BC. Overall, W-BC showed beneficial impacts for inflammation and fasting glucose but not serum lipids. The effect with hsCRP appears time dependent and indicated that the impact of W-BC on inflammatory markers may take up to 3 weeks to manifest, with the effect significant and durable for up to 9 months with continued use.

The results of this study agree with, and extend, prior research on W-BC related to inflammation. Studies in patients with arthritis showed that W-BC helped reduce inflammatory markers such as IL-6, tumor necrosis factor, and serum CRP [16-18]. Similar anti-inflammatory benefits from W-BC have been observed in studies of men with overweight and obesity, as well as postmenopausal women with type 2 diabetes [10-12]. While prior studies provided a groundwork for the acute anti-inflammatory effect of W-BC in populations who already suffer from chronic inflammation, this study sought to extend the generalizability of such an anti-inflammatory effect to healthy individuals without specific disease conditions.

To the best of our knowledge, there is only 1 other study that specifically examined healthy individuals and long-term durability of the anti-inflammatory effect with W-BC. Lubkowska et al [19] measured cytokine levels in healthy men for up to 6 weeks after W-BC and found a beneficial association between W-BC and serum cytokines. Their results, along with the current findings, demonstrate that W-BC can decrease inflammation in healthy adults, both in the short term (ie, 6 weeks) and the long term (ie, up to 9 months).

The current findings also add a durable clinical marker of systemic inflammation associated with future disease risk (hsCRP) to the cytokines previously studied. This is key because cytokines alone may be less accurate biomarkers than CRP for disease risk due to issues with stability, half-life, and serum variability. For example, IL-6, one of the main cytokines analyzed by Lubkowska et al [19, shows distinct diurnal variation and therefore time of day can be a confounder [20,21]. In contrast, CRP shows no diurnal variations lending it stability and reproducibility for comparison [22]. CRP may also be a better overall marker of disease risk by not only showing acute inflammation but also having persistence and stability in plasma. Research in cardiovascular disease indicates that hsCRP may be a better marker for future diagnoses of hypertension, myocardial infarction, and coronary heart disease [23,24]. Together, the current findings and prior outcomes elucidate potential mechanistic and clinical implications of short- and long-term W-BC.

Fasting glucose was also beneficially impacted by W-BC in this study, though the exact relationship is less clear. At 1 month, the association between W-BC and fasting glucose trended downward (*P*<.10). Durability analysis then revealed significant associations between W-BC and decreased fasting glucose for 2- and 3-month windows (adjusted *P*<.05). After 3 months, no linear relationship was found. Thus, the effect of W-BC on fasting glucose may be more time-constrained such that some benefits are seen for intermediate time windows but may not be durable chronic adaptations. This time-dependent interpretation is further supported in light of the lack of change seen in fasting glucose from the start to the end of the 9-month study. Interestingly, a very small increase in HbA_1c_ was noted at the final follow-up period (*P*<.01). However, the change was quite minimal (difference=0.13%) and within normal ranges at both time points (5.12 [0.27%] and 5.25 [0.29%], respectively) and not considered clinically significant.

From a clinical perspective, the current results extend inflammatory effects of W-BC to known markers of metabolic dysregulation. Specifically, decreases in blood glucose are known to modulate inflammation [25]. Thus, the beneficial impact on inflammation seen from W-BC may be achieved at least in part due to reductions in blood glucose. Indeed, the study by Wiecek et al [12] noted decreases in both blood glucose and CRP. While the previous study specifically examined women with type 2 diabetes and therefore elevated glycemia, the participants in this study were healthy, nondiabetic individuals. Thus, these results further extend the beneficial impact of W-BC on fasting glucose to those without an existing hyperglycemic diagnosis. Further targeted research would be needed to establish a definitive connection between the glucose modulating effects of W-BC and its anti-inflammatory benefits.

No significant effect from W-BC was found for the lipid markers of metabolism in either the comparison of study start to end or the linear modeling for specific time windows. This result slightly contradicts prior research finding a benefit of W-BC for lipids. In particular, Lubkowska et al [26] found that W-BC combined with exercise as a program to treat overweight resulted in improved lipid metabolism including decreased LDL and triglycerides and increased HDL. However, this study included exercise in a protocol targeted at treating overweight, thus applicability for W-BC specifically is less clear. A more recent study investigating lipid metabolism in healthy adults demonstrated that daily W-BC was associated with decreased LDL and triglycerides and increased HDL [27]. However, this study was analyzing acute effects immediately after daily application of W-BC while the present study, which found no significant effect on lipid metabolism, is focused on longer-term impacts. Thus, it is plausible that W-BC could impact lipid metabolism in the short term while not having a lasting durable benefit. It is also possible that a true effect exists, but this study was simply underpowered to discern it.

### Limitations

One major limitation of this study is sample size. Both the number of participants and the number of follow-up visits were small. To correct for any time-based confounding, time has been included in the models; however, other possible sources of confounding could exist. In addition, there was no control group. In general, control groups for W-BC are difficult since a participant cannot be blinded to whether they receive cryotherapy. In addition, this study was conducted in a free-living real-world environment rather than a controlled clinical setting. Such a design lends both benefits and drawbacks. The main drawback of this free-living environment is that external factors could lead to possible confounding. For example, weight loss in individuals with obesity has been linked to a decrease in inflammatory markers including CRP [28,29]. As weight loss information was not available, time was included as a proxy for any weight loss or other baseline improvements in health that participants may have experienced. However, time alone likely does not capture all confounding effects and thus the impact of external uncontrolled factors cannot be ignored.

In spite of the limitations, a free-living study also has unique benefits. In particular, such a design enables immediate applicability of results to real-world environments. This study did not involve a clinical protocol necessitating strict compliance, yet the results still demonstrated a significant benefit from recreationally participating in cryotherapy. Thus, the takeaways from these results may be applied more generally to the public in that individuals can simply incorporate cryotherapy into their daily lives.

Future directions of study could include analysis of dosage for optimal effect. For example, if the benefit of increased W-BC sessions plateaus at some point, then individuals can limit unnecessary time, expense, and exposure by participating in only an optimal number of W-BC sessions. Dosage could also be dependent on the durability of the effect. Given the rapidly rising popularity of cryotherapy, high-quality randomized controlled trials are needed to investigate these more specific outcomes.

### Conclusions

This study explores the impact of W-BC on healthy participants outside of any specific exercise protocol or disease state. In doing so, W-BC has been demonstrated to be potentially beneficial to the general population as an anti-inflammatory treatment. Furthermore, the anti-inflammatory effects show lasting durability for impact on future chronic inflammation. Finally, this study demonstrated that W-BC may have a beneficial, though time-constrained, impact on glucose.

## Abbreviations

CRP: C-reactive protein
HbA1c: hemoglobin A1c
HDL: high-density lipoprotein
hsCRP: high-sensitivity C-reactive protein
IL-6: interleukin-6
LDL: low-density lipoprotein
W-BC: whole-body cryotherapy

## Data Availability

The datasets generated during and/or analyzed during this study are available from the corresponding author on reasonable request.
